# Avoiding maternal-child death in DR Congo through infection control

**DOI:** 10.1186/1753-6561-5-S6-O21

**Published:** 2011-06-29

**Authors:** S Wembonyama

**Affiliations:** 1 University of Lubumbashi, Lubumbashi, Congo, Democratic Republic of the

## Introduction / objectives

In sub-Saharan Africa, 1 in 22 women is likely to experience infection or even death in child birth. Along with Nigeria and Ethiopia, the DRC is among the worse off. Hospital acquired infections(HAI) are a neglected and yet crucial factor in maternal mortality. Our objectives were to identify the role of HAI in the mortality observed in Lubumbashi maternal wards and to offer some answers to ensure patient safety.

## Methods

Data were collected from two maternity hospitals in the city of Lubumbashi, one tertiary level with 30 births and one secondary level maternity ward with 10 deliveries per day.

The following parameters were selected for study: maternal mortality rates, hospital hygiene, quality of care, performance of biomedical laboratories, supplies of antibiotics.

## Results

Maternal mortality is higher in the tertiary level maternity. HAI are a very prominent cause of maternal mortality, especially after a caesarean section.

Working conditions are the same in both hospitals as regards equipment. The level of skills is higher in the tertiary level maternity, but the sanitary conditions are appalling: no access to water, no antiseptics or disinfectants. Blood transfusions, infusions, injections are mostly carried out in extreme emergency, without basic safety precautions. Some deliveries are practiced with bare hands. Caesarean sections are performed without observance of asepsis.

## Conclusion

Progress are difficult because of the disorganization of the health system, lack of motivation of health personnel, the paucity of hospitals.A sensibilization of the general population locally and of international partners on the issue of safety in maternal child care is urgent. We need to mobilize partners as well as the public is needed to place emphasis on infection prevention and control through women organizations, patient groups and the media.

## Disclosure of interest

None declared.

## Note

This abstract was also presented as Poster P396.

